# NIST Materials Properties Databases for Advanced Ceramics

**DOI:** 10.6028/jres.106.055

**Published:** 2001-12-01

**Authors:** R. G. Munro

**Affiliations:** National Institute of Standards and Technology, Gaithersburg, MD 20899-8520

**Keywords:** ceramics, database, evaluated data, materials properties, mechanical properties, physical properties, structural ceramics, superconductivity, thermal properties

## Abstract

The NIST Ceramics Division maintains two databases on the physical, mechanical, thermal, and other properties of high temperature superconductors and structural ceramics. Crystallographic data are featured prominently among the physical property data and serve several important functions in the classification and evaluation of the property values. The scope of materials, properties, and data evaluation protocols are discussed for the two databases.

## 1. Introduction

Crystallography plays a central role in classifying and understanding the physical behavior of solid materials. It is for that reason that crystallographic data form an integral part of the data sets developed for the NIST materials properties databases for advanced ceramics.

The two general classes of materials considered in this work are structural ceramics and high temperature superconductors. These materials form two distinct types of advanced ceramics that have shared a common dilemma: significant economic benefits have been anticipated, but advances in applications of the materials have been slow to accrue. Uncertainties regarding the reliability of property values have contributed to the slowness of the progress, both with respect to developing new applications and to refining the characteristics of the materials. The NIST ceramics data program [1,2] is pursuing a three- tiered approach to assist in the resolution of this problem. At the root of the effort is the basic collection and organization of property data into more readily accessible and usable formats. Once collected, the data are evaluated with respect to how the materials were prepared and how the properties were measured. Inevitably there are apparent discrepencies among the reported results, and a significant portion of the NIST effort is devoted to understanding those differences in values. The latter effort involves models of property relations as well as statistical comparisons and analyses.

The evaluation of the data is particularly important in addressing the issues of two obvious, but extremely different, situations in data management: a lack of data and an abundance of data. Consider, for example, the case of high temperature superconductors (HTS). The interest in HTS materials [3] is motivated by their threefold potential for impact in science, technology, and the economy [4]. Scientifically, theories of conventional superconductivity do not appear to be adequate to explain the HTS phenomena, so new insights into material behavior are essential. Technologically, the production of an intense magnetic field using a superconductor with a high critical temperature (*T*_c_) may yield new processing control techniques for other advanced materials. Economically, nondestructive scientific measurement devices, superefficient motors, and high speed computers may soon evolve. In view of such potential utility, more than 50 000 technical papers have been published since the discovery of the HTS phenomena in 1986. Those papers are concerned predominantly with the production, characteristics, and properties of the HTS materials. This rather enormous amount of information makes access to the needed property data a somewhat daunting exercise. Fortunately, like other areas of forefront research, the focus of the HTS effort is on the central characteristics of the phenomenon, i. e., the critical temperatures, current densities, and field strengths, along with the structure of the materials. Other properties, particularly the thermal and mechanical properties [5], are studied also but appear more obscurely in the literature. The latter properties, however, are essential to the development of practical applications of the materials. Thus, for some properties, there is a need to consolidate large amounts of data, while for other properties, there is a need to assess the reliablity of limited amounts of data.

The emphasis on data evaluation leads rather naturally to the need for crystallographic data. The anisotropy of any physical property of a single crystal must be consistent with the observed symmetry in the physical structure of the crystal. By extension, anisotropy in the properties of polycrystalline materials should be correlated with the degree of texturing or particle alignment in the sintered bodies. Both crystal structure and texturing have important consequences for the behavior of materials subjected to external stimuli (temperature, pressure, and electromagnetic fields). Furthermore, structural data from crystallographic studies can be used to determine the coefficients of thermal expansion which, in turn, can be used in the evaluations of axial and volumetric derivatives of physical properties. Consequently, crystallographic data are indispensable in classifying and understanding the physical behavior of the materials, and these data help to form a basis on which to study and pursue the development of new materials.

## 2. Materials and Properties

To be an effective resource, the scope of the information system must be consistent with the needs and interests of both the materials research community and the application designers. At the same time, the enormous body of data must be condensed to a more manageable and reliable subset. A balance, then, is sought between all that is available and all that is needed.

That balance and the scope of the information system are influenced by three basic considerations: what materials should be covered; what properties are essential; and what measurement conditions are most relevant? Of these considerations, the first two are relatively straightforward, while the third one is a source of much concern.

The approach taken in the current NIST effort to provide evaluated data for ceramics gives higher priorities to materials with greater potential for commercial applications or for advancing the scientific understanding of the materials and their properties. For HTS materials, for example, it is recognized that any potential application of these materials must anticipate the necessity of maintaining the material in a superconducting state over long periods of time. Therefore, the technology of cryogenics is a critical factor in determining what will be commercially viable. Fortunately, the technology for sustaining liquid nitrogen in a closed-cycle, regenerative system is well developed, so that it is relatively routine to maintain a superconducting material at a temperature of 77 K, even on an industrial scale of operation. This consideration suggests that the materials with the greatest commercial interest should have *T*_c_ >77 K.

For structural ceramics, the application environment is also a principal consideration in selecting a material. The requirements for these materials often include dimensional and mechanical stability at very high temperatures (greater than 1000 °C) and durability in harsh environments. Thus, attention is usually directed towards advanced ceramics that retain their strength and hardness at high temperatures or that exhibit significant wear resistance.

Overviews of the materials and properties in the NIST databases on high temperature superconductors (HTS) and structural ceramics (SCD) are given in Tables 1 and 2. In each case, an attempt is made to provide a comprehensive range of properties. However, the distribution of data within the two collections differs according to the focal interest of the material classs. For superconductors, that interest is centered on the critical temperture (*T*_c_) and the critical current density (*j*_c_) and the associated properties needed to understand the critical behavior.

The majority of the HTS materials are oxide superconductors, but a useful range of the newer borocarbide superconductors is also included. For many of the mate rials in Table 1, the only property data available are the basic superconductor characteristics, such as the critical temperature. Because of the nonstoichiometry of HTS materials, it is not unusual for the lattice parameters at room temperature and pressure to be reported as part of the HTS material identification data. Indeed, oxygen content and lattice parameters often are sufficiently well correlated that the measurement of one can be used to make an empirical estimate of the other based on the observed correlation.

In contrast, studies on structural ceramics tend to focus on the mechanical properties of the materials, flexural strength being, by far, the most frequently mea sured property. For design purposes, mechanical and thermal properties are desired, not only as functions of temperature, but also as functions of bulk physical characteristics such as density, mean grain size, and porosity.

## 3. Data Evaluation

Perhaps the most important consideration of a reference database is the reliability of the data. The unpleasant consequences of unreliable data can be all too readily imagined. Nevertheless, there is often a struggle between the goal of having the best data and the quandary of having no data at all. In the HTS and SCD data efforts, therefore, the development of a database is viewed as a dynamic process in which the data set is continuously subject to refinement. In this process, an effort is made to ensure the reliability of the data, while treating all data as potentially valuable [6]. The resulting compromise is to use the best *available* data and to assign to each data set an indicator, called the data evaluation level, Table 3, to apprise the user of the current status of the data.

**Fig. 1:**
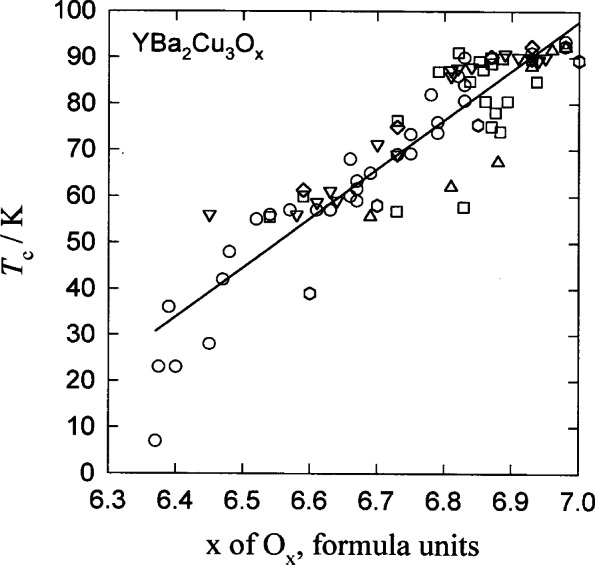
Data for critical temperature, *T*_c_, for YBa_2_Cu_3_O*_x_* with respect to oxygen content, without constraints on the processing of the material or its chemical or microstructural composition [7].

There are four stages of data evaluation, Table 4, that may be distinguished. The stages are progressive, each stage having useful results and subsequent stages build ing on preceding stages. The basic effort, Stage I, involves identifying and gathering the information that is available. While the identification of suitable sources of data involves some degree of judgement, the selectivity applied is more a matter of relevance than of merit. This effort is greatly facilitated by the use of computerized databases of titles and abstracts that can be searched for keywords. Upon the completion of Stage I, it is immediately possible to make comparisons of data and to obtain useful deductions about the materials and properties. Figure 1 shows a typical situation in which the critical temperature, *T*_c_, for YBa_2_Cu_3_O*_x_* (Y:123) is examined with respect to oxygen content [7]. In constructing this plot, no constraint regarding the processing of the material or its chemical or microstructural composition was applied. The resulting plot, not surprisingly, exhibits a considerable amount of scatter among the data points. Nevertheless, it is already clear that the value of *T*_c_ depends on the oxygen content, and, in this figure, the dependence is roughly linear.

**Fig. 2:**
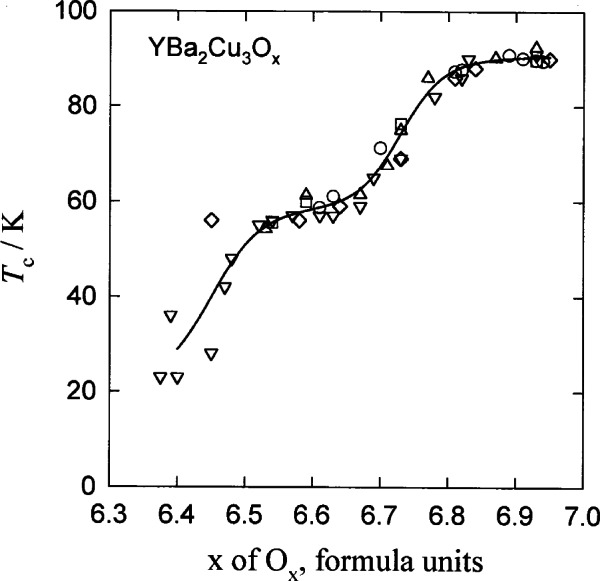
Data in Fig. 1 filtered to include only data from single phase, homogeneous specimens.

By design, Stage II data evaluation is more discriminating than Stage I. In Stage II, attention is given specifically to the processing and composition data for the material and to the descriptions of the measurement procedures. Figure 2 illustrates how significant details can be revealed by such restrictions. For Fig. 2, the data in Fig. 1 were filtered such that only data appropriate to single phase, homogeneous specimens were used. The-clearly reproducible two-plateau characteristic of *T*_c_ vs *x* for Y:123 is readily apparent.

**Fig. 3:**
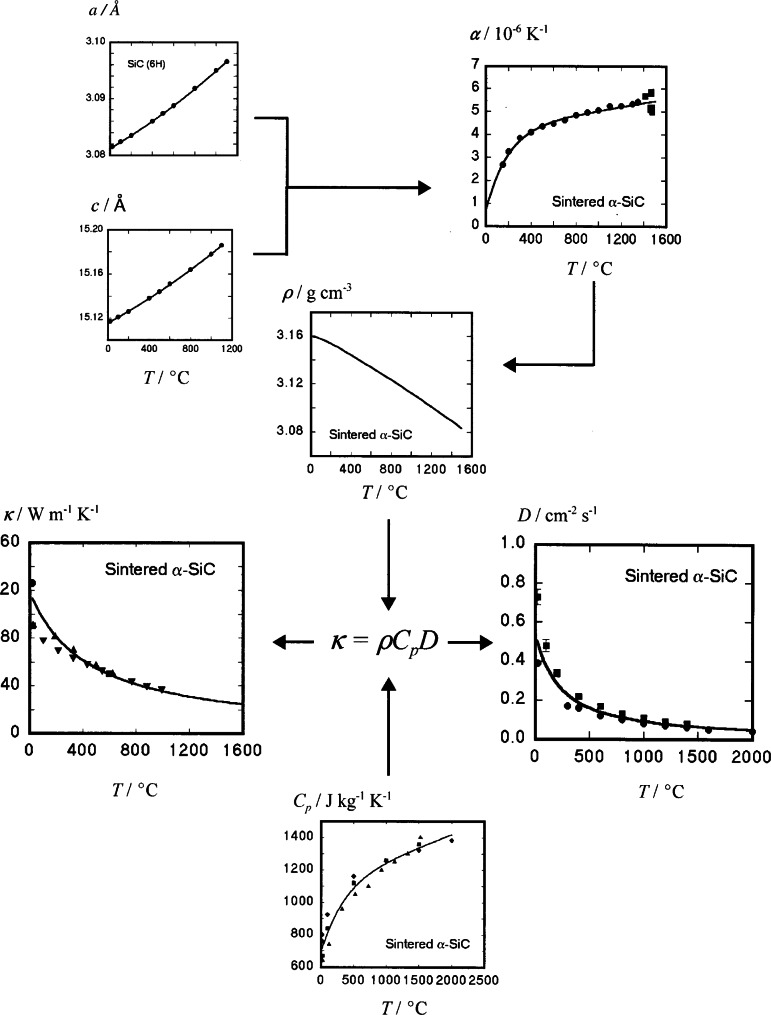
Data optimization through property relations, illustrating the use of multiple data sets to optimize the correlation of thermal conductivity and diffusivity for sintered a-SiC [8].

The modest goal of the data evaluation in Stage II is to ensure that the materials, measurements, and results are reasonable. In Stage III, the data are examined more closely for consistency among related properties. In this stage, the emphasis is on the property relations rather than the individual measurements of the properties. For example, thermal conductivity (*κ*) and thermal diffusivity (*D*) should be correlated through a relation involving the density (*ρ*) and the specific heat at constant pressure (*C_p_*); i. e., *κ* = *ρC_p_D*. To apply this relation, data from several independent studies would be combined, as in Fig. 3, to determine optimized and mutually consistent representations of the properties and to estimate the uncertainties of the values [8]. In this example, the temperature dependence of the lattice parameters (*a* and *c*) determine the mean linear coefficient of thermal expansion (*α*) which, in turn, is related to the temperature dependence of the bulk density. This result, combined with the independently determined specific heat (*C_p_*), permits the correlation of thermal conductivity and diffustivity to be optimized in such a manner that the four constituent properties are simultaneously mutually consistent. The agreement between the data points and the smooth curves attests to the effectiveness of the procedure.

The highest level of evaluation effort, Stage IV requires the development of explicit material models along with models describing the interactions among the constituent component of the material. Crystallographic and microstructural data are highly desirable in this work. In one study on Y:123, for example, an examination was made of how the critical temperature varies as the crystalline structure is perturbed by a variation of the oxygen content or by the application of an external pressure [9]. A model involving the lattice parameters of the crystal structure, the relative atomic coordinates of the the constituent ions, and the distribution of valence electrons was considered in an attempt to describe the variation of *T*_c_ under hydrostatic pressure and, by extension, to predict the variation of *T*_c_ under uniaxial stress.

## 4. Conclusion

The HTS and SCD databases have been developed to serve as reference databases for the numeric properties of nonconventional superconductors and structural ceramics. Crystallographic data are utilitzed in these databases to serve several functions. The crystallographic data sets themselves are, of course, part of the reference information characterizing the materials. Conversely, crystallographic data may be used for purposes of identification such as when lattice parameters are specified as part of the search criteria. Crystallographic data are also used both in data evaluation efforts, to help ensure that only data from comparable materials are being analyzed together, and in developing or applying models of material behavior. Many physical properties depend significantly on the phase compositions of the constituent particles, the interface or grain boundary regions, and the possible surface layers. Similarly, the size, shape, and distribution of pores, which may be treated formally as a secondary phase, can have a dramatic influence on property values. Crystallographic data often provide the key to understanding how these atomic and microstructural features affect the bulk properties of the material.

## Figures and Tables

**Table 1:** Materials in the HTS and SCD databases

High temperature superconductors	
Class	Examples^a^
Oxides	Y-Ba-Cu-O, Bi(Pb)-Sr-Ca-Cu-O, … (>170 chemical families)
Borocarbides	Y-Ni-B-C, … (>25 chemical families)
Structural ceramics	
Class	Examples^b^

Borides	TaB_2_, TiB_2_, ZrB_2_, …
Carbides	B_4_C, SiC, TiC, diamond, …
Nitrides	AlN, BN, Si_3_N_4_, …
Oxides	Al_2_O_3_, BeO, MgO, mullite, SiO_2_, TiO_2_, ZrO_2_, …
Oxynitrides	sialon, silicon oxynitride, …

a HTS materials are summarized here by chemical family designations. For example, Y-Ba-Cu-O represents all the superconducting chemcial compositions of Y, Ba, Cu, and O. This family includes YBa_2_Cu_3_O*_x_*, YBa_2_Cu_4_O*_y_*, and Y_2_Ba_4_Cu_7_O*_z_*. Elemental substitutions yield different chemical families (e.g., Y-Ba-Cu-O and Y-Ba(La)-Cu-O). Further variations occur due to nonstoichiometry.

b SCD materials are summarized here by generic formulas. Variations include differing sintering aids, densities, porosities, and grain sizes.

**Table 2:** Principal properties in the HTS and SCD databases

Category	Examples
Physical	Crystallography, grain size, density, porosity
Mechanical	Elasticity^a^, strength, hardness, toughness, creep
Thermal	Conductivity, diffusivity, expansion, specific heat
Corrosion^b^	Rate, activation energy, products
Conduction^c^	*T*_c_, *j*_c_, *H*_c1_, *H*_c2_, resistivity, thermopower, Hall effect, susceptibility

a Elasticity tensor for single crystal specimens. Young’s, shear, and bulk moduli and Poisson’s ratio for polycrystalline materials.

b SCD only.

c HTS only. Critical temperature, current density, and magnetic field strengths are identified by a subscript c.

**Table 3:** Data evaluation levels

Designation	Comment
Certified	Standard reference values, specific to known production batches
Validated	Confirmed via correlations and models
Evaluated	Basic acceptance criteria satisfied
Commercial	Manufacturer’s data for specific commercial materials
Typical	Derived from surveys of nominally similar materials
Research	Preliminary values from work in progress
Unevaluated	All other data

**Table 4:** Stages of data evaluation

Stage	Scope	Evaluation level	Comment
I	Data Collection	Unevaluated	Publicly accessible data
II	Basic Criteria	Evaluated	Material and measurement specifications; documentation; comparisons
III	Relational Analysis	Validated	Correlations and property relations; inter-polation and value estimates possible
IV	Modeling	Validated	Synthesis of data and theory; predictive capability
